# Management of kyphoscoliosis patients with respiratory failure in the intensive care unit and during long term follow up

**DOI:** 10.1186/2049-6958-7-30

**Published:** 2012-09-21

**Authors:** Nalan Adıgüzel, Zuhal Karakurt, Gökay Güngör, ÖzlemYazıcıoğlu Moçin, Merih Balcı, Cüneyt Saltürk, Feyza Kargın, Huriye Berk Takır, Ayşem Güven, Tülay Yarkın

**Affiliations:** 1 Respiratory Intensive Care Unit, SB Süreyyapaşa Chest Diseases and Thoracic Surgery Teaching and Research Hospital, Istanbul, Turkey; 2 Pulmonary Department, Erzurum Teaching and Research Hospital, Erzurum, Turkey

**Keywords:** Kyphoscoliosis, Intensive care, Long-term noninvasive ventilation

## Abstract

**Background:**

We aimed to evaluate the ICU management and long-term outcomes of kyphoscoliosis patients with respiratory failure.

**Methods:**

A retrospective observational cohort study was performed in a respiratory ICU and outpatient clinic from 2002–2011. We enrolled all kyphoscoliosis patients admitted to the ICU and followed-up at regular intervals after discharge. Reasons for acute respiratory failure (ARF), ICU data, mortality, length of ICU stay and outpatient clinic data, non-invasive ventilation (NIV) device settings, and compliance were recorded. NIV failure in the ICU and the long term effect of NIV on pulmonary performance were analyzed.

**Results:**

Sixty-two consecutive ICU kyphoscoliosis patients with ARF were enrolled in the study. NIV was initially applied to 55 patients, 11 (20%) patients were intubated, and the majority had sepsis and septic shock (p < 0.001). Mortality in the ICU was 14.5% (n = 9), reduced pH, IMV, and sepsis/septic shock were significantly higher in the non-survivors (p values 0.02, 0.02, 0.028, 0.012 respectively). Among 46 patients attending the outpatient clinic, 17 were lost to follow up and six were died. The six minute walk distance was significantly increased in the final follow up (306 m versus 419 m, p < 0.001).

**Conclusions:**

We strongly discourage the use of NIV in the case of septic shock in ICU kyphoscoliosis patients with ARF. Pulmonary performance improved with NIV during long term follow up.

## Background

Kyphoscoliosis (KS) is characterized by diminished chest wall compliance and impaired respiratory mechanics, leading to progressive hypoventilation, hypercapnia and chronic respiratory failure (CRF) [1]. Acute exacerbations, particularly respiratory tract infections, rapidly worsen these patients’ respiratory conditions and precipitate acute respiratory failure (ARF), which usually requires intensive care unit (ICU) hospitalization and either non-invasive or invasive mechanical ventilation [2].

Non-invasive, positive pressure ventilation (NIV) has become an accepted treatment option for CRF in chest wall diseases [3]. Following the use of NIV in kyphoscoliosis patients with CRF, improvements in hypoventilation symptoms and a reduction in hospital readmissions have been shown [4-6]. However, ICU management and the long-term, post-ARF/ICU outcomes are rarely reported in these patients.

In the present study, we aimed to evaluate ICU management and long-term outcomes of patients with CRF due to kyphoscoliosis.

## Methods

This study was conducted in a level III medical intensive care unit at a tertiary education hospital. The study was designed as a retrospective observational cohort study between August 2002 and May 2011. Patients were followed up for at least 12 months after discharge from ICU. The study was approved by the local ethical committee of the government teaching hospital.

### Patients

Kyphoscoliosis patients who were admitted to the medical ICU with acute or acute-on-chronic hypercapnic respiratory failure were enrolled in the study. ARF was defined by the presence of acute breathing discomfort and arterial blood gases values (ABG): partial arterial oxygen pressure (PaO_2_) < 60 mmHg on room air, or PaO_2_ over the fraction of inspired oxygen (PaO_2_/ FiO_2_) <300, partial arterial carbon dioxide pressure (PaCO_2_) ≥ 45 mmHg and pH ≤ 7.35 [7]. The reasons for ARF were recorded as cor pulmonale [8], sepsis/septic shock [9] due to pneumonia, lower respiratory tract infection, urinary tract and blood stream infection.

Sepsis was defined as proven or strongly suspected infection, and two of four criteria for systemic inflammatory response syndrome: 1. A respiratory rate more than 20/min or partial carbon dioxide pressure less than 32 mmHg; 2. A heart rate of more than 90 beats/min; 3. A temperature more than 38.3°C or less than 36.0°C; 4. A white blood cell count of more than 12,000 cells/μl or less than 4,000 cells/μl, or more than 10% immature cells [9]. Septic shock was defined as sepsis-induced hypotension (systolic blood pressure of less than 90 mmHg or a reduction of more than 40 mmHg from baseline in absence of other causes of hypotension), and patients requiring vasopressor to maintain a mean arterial pressure of more than 70 mmHg despite adequate fluid resuscitation [9].

Pneumonia was defined as the acute onset of symptoms suggestive of a lower respiratory tract infection and radiographical evidence of a new infiltrate [10]. The presence of a nosocomial infection on admission to ICU was recorded, and all patients were treated according to guidelines [11].

### Assessment of mechanical ventilations

#### Non-invasive mechanical ventilation

NIV was delivered in pressure assist-control mode with ICU mechanical ventilators via a double tube circuit with a full-face mask. Pressure support (PS) was initially set at 10 cmH_2_O and gradually increased to a maximum of 30 cmH_2_O until the exhaled tidal volume was 8-10 ml/kg and guided by patient tolerance. Positive end-expiratory pressure (PEEP) was set at 5 cmH_2_O and raised if needed to treat hypoxemia, or lowered to enhance patient comfort. The fraction of inspired oxygen (FiO_2_) was adjusted to keep oxygen saturation (SaO_2_) at 90%. NIV was applied intermittently for periods of 1 to 4 h and initial ABG samples were obtained at the end of first hour. The duration of each session was determined by improvement in ABG levels, the level of consciousness, and patient compliance. The level of consciousness was assessed by the Glasgow coma scale (GCS) [12].

#### Intubation and NIV failure criteria

NIV was considered to have failed if at least one of the following criteria for endotracheal intubation (ET) occurred: 1. Cardiac arrest or severe hemodynamic instability (mean arterial pressure < 65 mmHg or the need for vasoactive agents); 2. Respiratory arrest; 3. Mask intolerance with agitation requiring sedation; 4. Severe difficulty clearing secretions; 5. Failure of gas exchange within the first 1 to 4 h of NIV therapy; 6. Lack of improvement in consciousness [3,13].

#### Invasive mechanical ventilation

Invasive mechanical ventilation (IMV) was delivered in pressure assist-control ventilation (PCV) mode and PS was initially set at 10 cmH_2_O and then gradually increased to a maximum of 30 cmH_2_O until the exhaled tidal volume was 8-10 ml/kg, as with the NIV settings. An intermittent sedation protocol was applied with midazolam according to Richmond agitation-sedation scale [14]. After cessation of sedation, patients were evaluated for weaning. When patients were conscious and hemodynamically stabile, a T-tube trial was performed, and after 30 minutes patients were extubated according to our weaning protocol. If there was still a demand for mechanical ventilation after weaning due to hypercapnia we preferred to continue with NIV. We prescribed domiciliary NIV devices according to home mechanical ventilation guidelines after a good response was achieved by NIV during the ICU stay [15]. All patients received education from a specialized NIV nurse for using these NIV devices prior to discharge.

### Follow up

Patients were evaluated for the efficacy of NIV and treatment adherence in an outpatient clinic one month after ICU discharge, and then at 3–6 months intervals. Treatment efficacy was assessed by clinical status and ABG values. IPAP (inspiratory positive airway pressure) and EPAP (expiratory positive airway pressure) values of NIV devices were recorded. The physiological and functional parameters were compared with IPAP-EPAP and IPAP-EPAP/weight as defined by Budweisser et al. [16]. Objective NIV usage was also measured by a specialized NIV nurse at every outpatient clinic visit by checking the built-in time counter of the NIV device. The NIV compliance was defined by the use of NIV more than four hours a day or > 20 hour a week [15].

### Data collections

We recorded patients’ age, gender, body mass index (BMI, kg/m^2^), Glasgow coma scale (GCS) [8], acute physiology and chronic health evaluation (APACHE) II scores [17], ABG values on admission to ICU, co-morbid diseases, causes of ARF, presence of oxygen therapy, NIV or IMV (tracheostomy) use for long-term home therapy prior to ICU stay, ICU respiratory management (need for NIV, ventilation mode and duration, need for and duration of tracheal intubation, tracheostomy), status on ICU discharge (deceased or alive) and respiratory status/management on ICU discharge (spontaneous breathing, NIV, tracheostomy).

Arm span (largest distance across the middle fingers when the arms are stretched horizontally sideways) was used as the height of KF patients for BMI and spirometry measurements [18]. The six-minute walk test (6-MWT) was performed on eligible patients in the follow up period, and the 6-minute walk distance (6-MWD) was recorded (in meters) [19].

We also recorded pulmonary function tests, ABG values at the first and last outpatient clinic admission, treatment compliance, and whether the patient was deceased or surviving. The total number of hospital and ICU readmissions with additional ARF episodes were obtained from the hospital electronic database.

### Statistical analysis

A descriptive analysis was used for patients’ demographics, ICU data. Survivors versus non-survivors and NIV success versus NIV failure were compared by the Mann Whitney *U* test for non-parametric continuous variables, and the *χ*^2^ test was used for dichotomous variable. The relationship of IPAP-EPAP/weight with 6-MWD and PaCO_2_ and spirometry results were evaluated with the Spearman’s correlations test. The Wilcoxon two related sample test was used for comparison of the first and last ABG and spirometry test results during the follow up period. Median and interquartile ratio was used for continuous variables. Count and percentage were used when applicable. A p value < 0.05 was accepted as statistically significant.

## Results

### Patients

During the study period, 62 kyphoscoliosis patients (29 female) with ARF were included. Thirty-nine patients were admitted from the ward, 17 from the emergency department, and six from another ICU (Table 1). The reasons for kyphoscoliosis were idiopathic (n = 42, 67.7%), post-rachitic (n = 9, 14.5%), rheumatologic disease (n = 7, 11.4%), post-poliomyelitis (n = 2, 3.2%) and post-traumatic (n = 2, 3.2%). The median (interquartile range) age was 52 (45–65) years, and median BMI was 23 (21–27) kg/m^2^. All patients were hypercapnic and 55 (88.7%) patients were both hypercapnic and hypoxemic. The reasons for ICU admission were: two patients (3.3%) had a home NIV device problem, 26 (41.9%) had cor pulmonale, 26 (41.9%) had severe sepsis, and eight (12.9%) had septic shock. Pneumonia was diagnosed in 17 patients, seven of which had septic shock. Other reasons for sepsis were urinary tract infection (n = 3), lower respiratory tract infection (n = 13), and blood stream infection (n = 1). Patients’ comorbidity and domiciliary devices (NIV or oxygen), and ABGs values on admission to the ICU are shown in Table 1.

**Table 1:** Patient’s comorbidity and ICU data

**Pre ICU localization, n (%)**	
ward	39 (62.9)
Emergency department	17 (27.4)
Other ICU	6 (9.7)
**Comorbid diseases, N (%)**	46 (75.4)
Diabetes mellitus	4 (6.6)
Hypertension	20 (32.3)
Congestive heart failure	14 (22.6)
Renal failure	1 (1.6)
**Domiciliary oxygen therapy, N (%)**	15 (24.6)
**Domiciliary ventilator therapy, N (%)**	4 (6.5)
**APACHE II on ICU admission, median(IQR)**	15 (12–19)
**Arterial blood gases on admission, median(IQR)**	
pH	7.34 (7.29-7.40)
PaCO_2_, mmHg	69.5 (61.0-81.7)
PaO_2_, mmHg	70.9 (54.3-84.2)
HCO_3_, mmol/l	37.7 (33.1-41.1)
PaO_2_/FiO_2_	192 (145–264)

ICU, intensive care unit; APACHE, acute physiology and chronic health evaluation; PaO_2_, partial arterial oxygen pressure; PaCO_2_, partial arterial carbondioxide pressure; FiO_2_, fraction of inspired oxygen; HCO_3_, hydrogen bicarbonate; IQR, interquartile ratio.

### Assessment of mechanical ventilation

**Figure 1:**
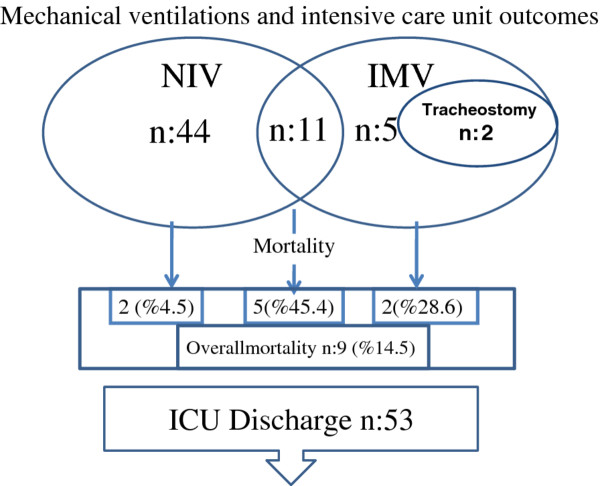
Mechanical ventilation outcomes of kyphoscoliosis patients in intensive care unit.

In the study period, seven patients (11.4%) initially received IMV (two via tracheostomy) and 55 (88.6%) patients received NIV. Eleven patients (17.7%) were intubated after initial NIV application. Two patients died during NIV therapy due to acute myocardial infarction. The overall NIV success rate was 76.4% (42/55). Mechanical ventilation and ICU outcomes are shown in Figure 1. Five NIV failures and two patients who were initially intubated died because of sepsis with a resistant pathogen.

All intubated and tracheostomized (n = 2) patients were ventilated in pressure assist-control mode with the median (IQR) PS 17 (10–19) cmH_2_O to get tidal volume 8–10 ml/kg and PEEP to 6 (5–6) cmH_2_O. During NIV applications, the median PS was 15 (10–19) cmH_2_O and PEEP was 6 (5–6) cm H_2_O; the median NIV hours per day were 11.0 (9.2-12.9). None of the patients had pneumothorax. The median length of stay in the ICU was 8 (5–14) days. The overall ICU mortality was 14.5% (see Figure 1 for mortality relative to mechanical ventilation support). Five patients were tracheostomized due to weaning failure. Surviving and non-surviving patients were further compared according to gender, age, initial ABGs, mechanical ventilation (IMV or NIV), and the presence of sepsis and septic shock on admission to the ICU (Table 2). The non-surviving kyphoscoliosis patients had significantly lower pH values, a higher rate of IMV, and a higher rate of sepsis and septic shock (Table 2) (p values were 0.02, 0.02, 0.028, 0.012 respectively). Both groups had a statistically similar length of stay in the ICU (p = 0.40) (Table 2).

**Table 2:** A comparison patient data of survivors versus non-survivors of kyphoscoliosis patients in the ICU

	**Survivors, n = 53**	**Non-survivors, n = 9**	**p**
Gender, male/female	29/24	4/5	0.72
Age, years	51 (45–64)	62 (45–70)	0.49
APACHE II, median(IQR)	15 (12–19)	16 (14–19)	0.26
pH on admission, median(IQR)	7.37 (7.30-7.40)	7.29 (7.28-7.31)	0.02
PaCO_2_ on admission, median(IQR)	69.0 (60.1-80.8)	80.0 (73.0-83.0)	0.10
PaO_2_/FiO_2_, median(IQR)	195 (147–275)	161 (145–189)	0.08
IMV, n (%)	11 (20.8)	7 (77.8)	0.02
NIV, n (%)	48 (90.6)	7 (77.8)	0.26
Sepsis on admission, n (%)	19 (35.8)	7 (77.8)	0.028
Septic shock on admission, n (%)	4 (7.5)	4 (44.4)	0.012
Length of stay in ICU, day	8 (5–14)	13 (8–18)	0.40

ICU, intensive care unit; IQR, interquartile ratio; APACHE, acute physiology and chronic health evaluation; PaCO_2_, partial arterial carbondioxide pressure; PaO_2_, partial arterial oxygen pressure; FiO_2_, fraction of inspired oxygen; IMV, invasive mechanical ventilation; NIV, non-invasive mechanical ventilation.

In the 55 patients who received NIV ABGs values were obtained on admission to the ICU and after the first hour of NIV, to assess NIV response (summarized in Table 3). NIV failure patients had significantly lower pH values at admission and after the first hour of NIV (p < 0.005, p < 0.026 respectively, Table 3).

**Table 3:** Arterial blood gases values on admission to the ICU and after the first hour of NIV in patients with NIV success and failure

	**NIV success, n = 44**	**NIV failure, n = 11**	**p**
APACHE II score, median(IQR)	14 (12–16)	20 (17–23)	0.001
GCS, median(IQR)	15 (14–15)	14 (10–15)	0.002
Respiratory rate, median(IQR) [bpm]	25 (22–31)	26 (19–37)	0.58
***ABGs on admission to the ICU, median(IQR)***			
pH	7.37 (7.30-4.40)	7.28 (7.26-7.32)	0.005
paCO_2_, mmHg	69.0 (61.2-78.6)	81.6 (72.5-93.2)	0.015
PaO_2_/FiO_2_	201 (149–274)	161 (137–191)	0.12
HCO_3_, mmol	37.7 (33.2-41.2)	38.9 (33.1 -41.1)	0.74
***First control ABGs after NIV, median(IQR)***			
pH	7.38 (7.34-7.42)	7.29 (2.25-7.40)	0.026
paCO_2_, mmHg	62.9 (55.6-77.7)	77.0 (57.8-90.6)	0.07
PaO_2_/FiO_2_	234 (202–293)	193 (137–219)	0.015
HCO_3_, mmol	35.4 (33.0-40.0)	36.4 (32.5-39.8)	0.74

ABG, arterial blood gas; ICU, intensive care unit; IQR, interquartile ratio; APACHE, acute physiology and chronic health evaluation; GCS, Glascow Coma Scale; bpm, breaths per minute; PaCO_2_, partial arterial carbondioxide pressure; PaO_2_, partial arterial oxygen pressure; FiO_2_, fraction of inspired oxygen; HCO_3_, hydrogen bicarbonate.

**Figure 2:**
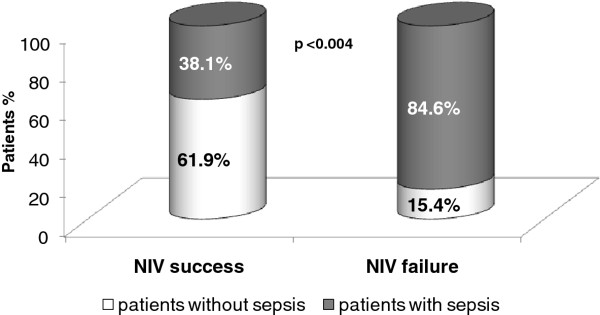
Sepsis in patients with non-invasive ventilation success and failure.

There were 13 patients where NIV failed; 11 were intubated for IMV and two died due to myocardial infarction during NIV. A significantly higher rate of sepsis/septic shock was found in the NIV failure patients (n = 11/13) than in the NIV success patients (n = 16/42) (p < 0.004, χ square, fisher exact test, Figure 2).

#### Follow up period

**Figure 3:**
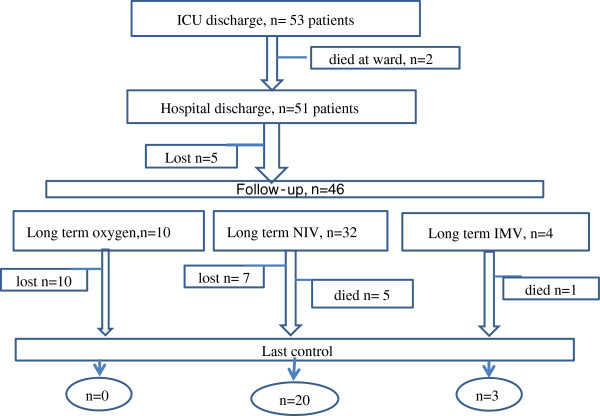
Follow-up outcomes of kyphoscoliosis patients.

Fifty-three patients were discharged from ICU (18 to home, 35 to the the ward). Two patients died in the ward and overall hospital mortality was 17.7% (n = 11). The long-term follow up outcomes are summarized in Figure 3.

After the initial one-month follow up at the outpatient clinic, 29 patients were followed-up for longer than 12 months. The median (IQR) follow up was 48 months (24–65) and NIV compliance rate was 79.3%. The re-hospitalization of eight of these 29 patients was recorded during the follow up period and they were re-admitted 19 times for pneumonia (n = 8), pulmonary embolism (n = 3), acute cor pulmonale (n = 5), pneumothorax (n = 1), and NIV device impairment (n = 2). The initial and final pulmonary function test results (ABG, spirometry and 6-MWD) are summarized in Table 4.

**Table 4:** Long-term pulmonary function tests of kyphoscoliosis patients

	**First control**	**Last control**	
	**Median (IQR)**	**Median (IQR)**	p*
pH	7.43 (7.41-7.44)	7.43 (7.40-7.46)	0.001
PaCO_2_	47.7 (44.0-53.9)	50.4 (46.3-54.0)	0.18
PaO_2_/FiO_2_	300 (275–332)	304 (269–328)	0.75
HCO_3_mmol	31.0 (28.6-35.3)	32.1 (30.1-34.8)	0.44
FEV_1_, ml	670 (550–860)	840 (520–940)	0.46
FEV_1_%	34 (18–40)	35 (21–44)	0.50
FVC, ml	830 (740–1100)	955 (640–1160)	0.35
FVC%	32 (21–38)	34 (22–41)	0.50
FEV_1_/FVC	82 (78–89)	88 (81–91)	0.87
6-MWD, m	306 (220–405)	419 (348–462)	0.001

Wilcoxon two related sample test. PaCO_2_, partial arterial carbondioxide pressure; PaO_2_, partial arterial oxygen pressure; FiO_2_, fraction of inspired oxygen; HCO_3_, Hydrogen bicarbonate, FEV_1_, forced expiratory volume in 1 second; FVC, forced vital capacity; 6-MWD, 6 minute walking distance.

Median IPAP and EPAP of NIV device were 20 (18–25) cmH_2_O, and 5 (5–6) cm H_2_O respectively in initial follow up. The median IPAP-EPAP difference was 15 (12–19) cmH_2_O, and the median IPAP-EPAP/weight ratio was 0.26 (0.16-0.31). The relationship between IPAP-EPAP/weight with 6-MWD, PaCO_2_ and spirometry results were evaluated with Spearman’s correlations test. IPAP-EPAP/weight ratio was significantly positively correlated with 6-MWD (r = 0.49, p = 0.029) but no correlation was found between PaCO_2_ and spirometry test results.

## Discussion

In the present study we evaluated acute and chronic respiratory failure management and outcomes in a substantial population of the rare disease kyphoscoliosis. The success rate of applying non-invasive mechanical ventilation in kyphoscoliosis patients with acute respiratory failure was found to be 76.4% in our ICU. The rate of sepsis in patients with NIV failure was higher than in patients with NIV success. The mortality rate was higher in patients in ICU with NIV failure, using IMV, and with septic shock. The prescription rates of long-term mechanical ventilation and NIV compliance were 70.6% and 79.3% respectively. The six-minute walk distance increased significantly after long-term NIV.

There are currently no reports on the reasons why kyphoscoliosis patients with acute respiratory failure require ICU admission and mechanical ventilation. However, pneumonia and cor pulmonale are seen in one third of hospitalized chronic obstructive pulmonary disease (COPD) patients with NIV [20]. The primary reasons for ARF in our kyphoscoliosis patients were cor pulmonale and sepsis. Pneumonia was the primary cause of sepsis. We found that NIV failure patients had a higher mortality rate than initially intubated patients. This may be related to delayed intubation due to a minimal improvement of ABG values after the first hour of NIV treatment. NIV success patients had a 4.5 percent mortality rate (Figure 1).

NIV failure risk factors in COPD are well studied and defined with guidelines [3,21-23]. Confalonieri and co-workers presented a chart where NIV failure in patients with COPD was in a red zone [23]. Patients in the red zone had a respiratory rate ≥ 30, GCS ≤ 11, pH < 7.25, and an APACHE II score ≥ 29 on admission to ICU. These were accepted as the most important parameters for NIV failure criteria [23]. Although the patients presented here were not diagnosed with COPD, NIV failure kyphoscoliosis patients in the ICU had a significantly higher APACHE II score and respiratory rate, but lower GCS and pH values (20 versus 14 score; 26 versus 25 breath /min, 14 versus 15 score; 7.28 versus 7.37, respectively). These results were not as severe as those observed in NIV failure due to COPD, as described [3,21-23].

### Follow up

NIV compliance for long-term usage varies according to the underlying diseases, such as COPD, obesity hypoventilation, neuromuscular disease and kyphoscoliosis. The NIV compliance rate of kyphoscoliosis patients is currently reported to be between 79 and 90% [24-26]. In the present study, 29 kyphoscoliosis patients with NIV were tightly compliant to the outpatient clinic controls. Patients without a NIV device (n = 10) did not attend the outpatient clinic. The NIV compliance rate (>4 hours a day) was 79.3%.

Volume or pressure cycled NIV settings have previously been used for long-term home therapy and both have showed similar results in various studies [3,27-29]. We used pressure cycled NIV devices. Budweiser and co-workers showed that PS greater than 15 cmH_2_O improved the decrease in PaCO_2_ for long-term follow up in patients with restrictive thoracic diseases [16]. They concluded that IPAP-EPAP/weight ratio correlated with the PaCO_2_ decrease at the initial follow up after hospital discharge with long-term NIV [16]. However we did not find any correlation between ΔPaCO_2_ and IPAP-EPAP/weight and the initial outpatient clinic data (pulmonary function, 6-MWD and ABG values). IPAP-EPAP/weight ratio was significantly correlated with 6-MWD but not with the other parameters. A recent review which evaluated 41 studies published over an 18 year period, looked at the physiological effects of NIV on work-of-breathing (WOB), pressure-support of 15 cmH_2_O and a PEEP of 5 cmH_2_O, and showed reduced WOB in patients primarily with chronic pulmonary disease including kyphoscoliosis [30]. In the present study IPAP was, in most cases, set greater than 20 cmH_2_O, and EPAP was set as 5 cmH_2_O, similar to previous studies [16,30].

The mortality rates of kyphoscoliosis patients with long-term domiciliary NIV therapy have been reported as 9.5% over two years, and 21% over five years [26,28]. In the present study the mortality rate was 20.7% over 4 years.

There are some limitations in our study. Firstly, we designed it retrospectively and in one center. Secondly, the physiological respiratory muscle function tests (ie, diaphragm functions) and the measurement of the degree of spinal curvature were not done. The use of the degree of curvature and the severity of respiratory failure relation is controversial [31,32]. Thirdly, the ICU mortality risk analysis was not done due to small number of non-surviving patients.

## Conclusion

Kyphoscoliosis patients with respiratory failure due to pump failure have a mainly good response to NIV. However, in specific disease groups and the presence of sepsis/septic shock NIV failure may result. For this reason we strongly discourage the use of NIV in the case of septic shock. Although this is a single center study and we cannot generalize the results for all patients, this study may help to make a decision on the clinical management of kyphoscoliosis patients with ARF in the ICU. Pulmonary function tests and 6-MWD of kyphoscoliosis patients can improve with nearly 15 cmH_2_O pressure support with pressure cycled NIV devices during long term follow up.

## Competing interests

As the authors of “Management of kyphoscoliosis patients with respiratory failure in the intensive care unit and during long-term follow up” we declare that we have no competing interests that might be related to the contents of the manuscript.
